# The complete chloroplast genome sequence of *Sedum bulbiferum* (Crassulaceae)

**DOI:** 10.1080/23802359.2022.2160220

**Published:** 2023-05-24

**Authors:** Zijie Deng, Kerui Huang, Peng Xie, Suisui Xie, Ningyun Zhang, Hanbin Yin, Mo Ping, Yun Wang

**Affiliations:** a Hunan Provincial Key Laboratory for Molecular Immunity Technology of Aquatic Animal Diseases, College of Life and Environmental Sciences, Hunan University of Arts and Science, Changde, China; b The First High School of Changsha, China

**Keywords:** Sedum bulbiferum, chloroplast genome, phylogenetic analysis

## Abstract

*Sedum bulbiferum* is a traditional medicinal plant in China, with few reports on its chloroplast genome. In this study, the chloroplast genome of *Sedum bulbiferum* was characterized, and its phylogenetic position among other closely related species was studied. The results showed that the full length of the chloroplast genome was 150,074 bp, containing a large single-copy (LSC) region and a small single-copy (SSC) region of 81,730 and 16,726 bp, respectively, as well as two inverted repeat regions (IRs) of 25,809 bp like other plants. A total of 128 genes were found, including 83 protein-coding genes, 37 tRNA genes, and eight rRNA genes. Phylogenetic analysis showed that *Sedum bulbiferum* is closely related to *Sedum emarginatum, Sedum alfredii, Sedum tricarpum, Sedum plumbizincicola,* and *Sedum sarmentosum.*

## Introduction

**Figure 1.**
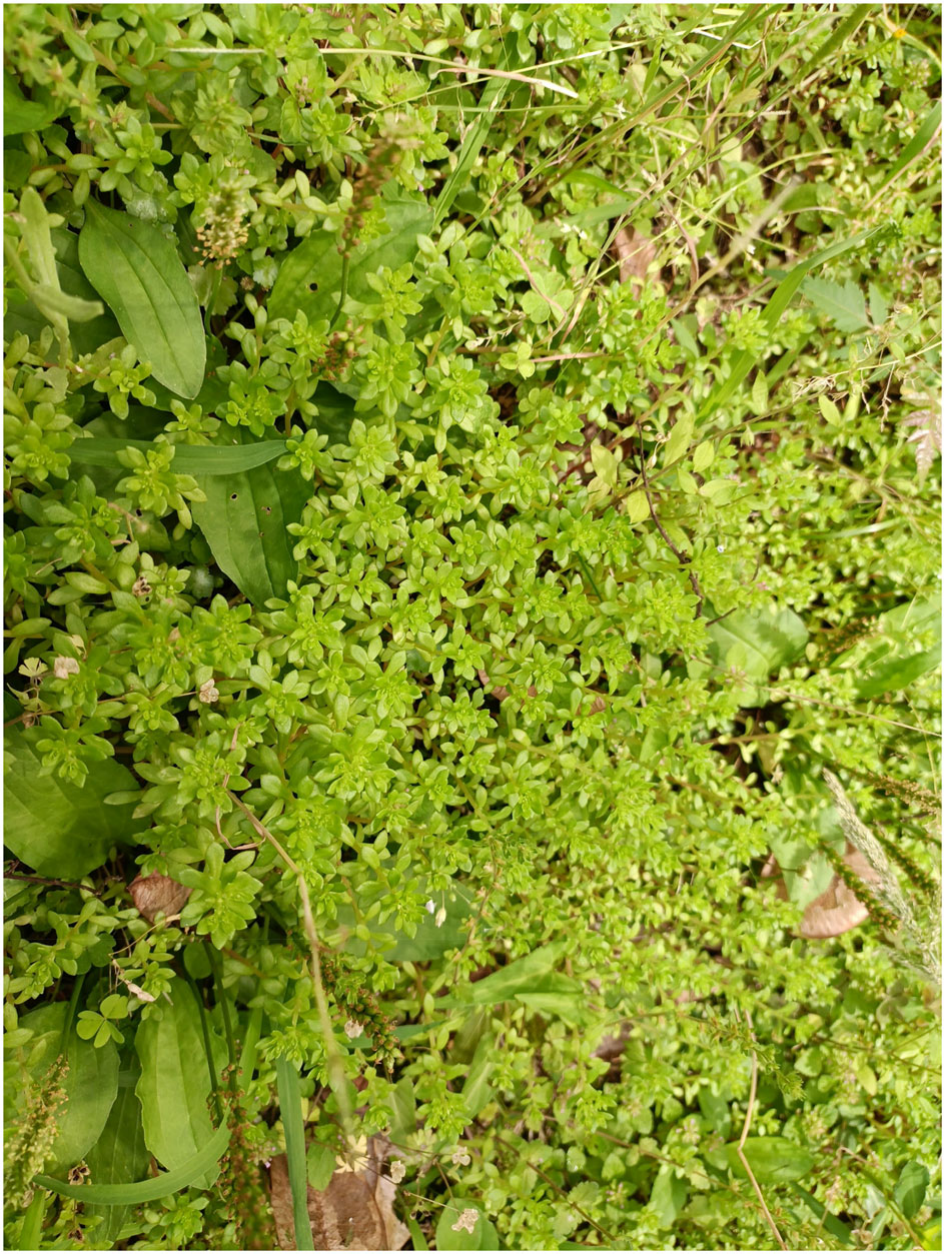
The picture of the collected sample of *S. bulbiferum.* The picture is self-taken by Zijie Deng at Changde Baima Lake Cultural Park, Changde, Hunan province, China (29°03′6.55ʺN, 111°39′59.54ʺE; 33 m). In the picture, *S. bulbiferum* is the wide-spread plant with small leaves, which has a fibrous root, 7–22 cm long stems, white globular bulbils that are viviparous, and a 3-branched and numerous-flowered cyme.

*Sedum bulbiferum* Makino 1891 is a perennial herb of the genus *Sedum* in the Crassulaceae. It has a fibrous root, 7–22 cm long stems, white globular bulbils that are viviparous, and a 3-branched and numerous-flowered cyme. The plant is widely distributed in Hunan, Hubei, Guangdong, Guangxi, and other Chinese provinces. It has grown in the Korean peninsula and Japan, preferring the shade of low mountains and flat trees below 1000 meters above sea level (Figure 1) (Wu and Raven 2001). After drying, *S. bulbiferum* has medical properties in traditional Chinese medicine culture, commonly used in China. It has been reported that *S. bulbiferum* has various pharmacological activities, such as treating malaria, rheumatism, and indigestion. Previously, it was also found that flavonoids isolated from other *Sedum* plants have significant antitumor biological activities (Meng et al. 2019). Previous reports on *S. bulbiferum* have mainly focused on its pharmacology. However, there are few reports on its evolution and classification, and its chloroplast genome is unknown. Therefore, the chloroplast genome of *S. bulbiferum* is sequenced and described in this study, and a phylogenetic tree based on the chloroplast genome is constructed to explore its phylogenetic status. This provides a theoretical reference for further phylogenetic research and other related research.

## Materials

For plant materials, five fresh leaves were obtained from *S. bulbiferum* cultivated in the Changde Baima Lake Cultural Park, Changde, Hunan province, China (29°03′6.55ʺN, 111°39′59.54ʺE; 33 m). Later, the voucher specimens were dried and preserved well at the College of Life and Environmental Sciences, Hunan University of Arts and Sciences (Contact Person: Kerui Huang, huangkerui008@163.com, voucher number ZYJT005).

## Methods

Healthy leaves were selected and dried to extract total genomic DNA using the DNeasy plant tissue kit (TIANGEN Biotech Co., Ltd., Beijing). Subsequently, the library was constructed, and the sequencing process was performed using an Illumina HiSeq 2500 platform (Shanghai Personalbio Technology Co., Ltd., China). After filtering out the low-quality reads using fastp (Chen et al. 2018), 67,498,968 reads were obtained. Then, the obtained reads were used for the *de novo* assembly of the *S. bulbiferum* chloroplast genome using GetOrganelle v1.7.5 (Jin et al. 2020) (Supplemental material 1). Finally, CPGAVAS2 (Shi et al. 2019) was used to annotate the chloroplast genome.

To determine the phylogenetic location of *S. bulbiferum*, a maximum-likelihood (ML) tree was constructed using IQ-Tree v1.6.8 (Chernomor et al. 2016) based on the chloroplast genome of *S. bulbiferum* and its related species. A total of 49 chloroplast genomes were obtained from GenBank. A total of 55 protein-coding genes shared by all genomes were found and extracted, then MAFFT v7.313 (Rozewicki et al. 2019) was used for separate alignment of each gene. Then, Gblocks 0.91 b was used for sequence masking of each gene, and end-to-end connections of the genes were used to form a supergene of each species (Guo et al. 2022). Maximum likelihood phylogenies were inferred using IQ-TREE (Nguyen et al. 2015) under the TVM + I + F model for 5000 ultrafast bootstraps and the Shimodaira–Hasegawa–like approximate likelihood-ratio test.

## Results

**Figure 2.**
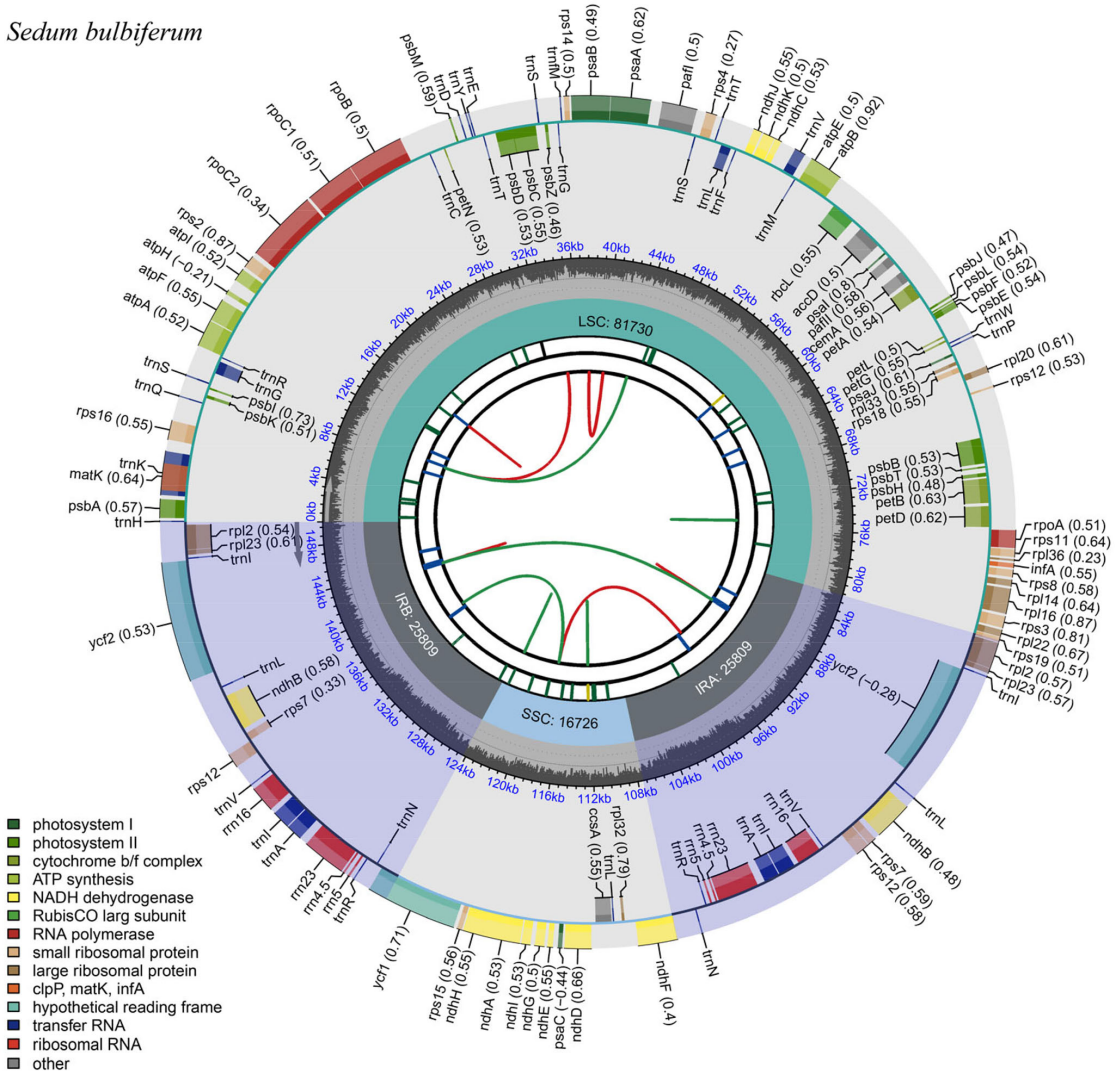
Gene map of the *S. bulbiferum* chloroplast genome. From the center outward, the first track indicates the dispersed repeats; The second track shows the long tandem repeats as short blue bars; The third track shows the short tandem repeats or microsatellite sequences as short bars with different colors; The fourth track shows small single-copy (SSC), inverted repeat (Ira and Irb), and large single-copy (LSC) regions. The GC content along the genome is plotted on the fifth track; The genes are shown on the sixth track.

The chloroplast gene structure of *Sedum bulbiferum* (Figure 2 and Supplemental material 2), as with most plants, is a circular molecule with a length of 150,074 bp that has a typical quadripartite structure comprising a large single-copy (LSC) region (81,730 bp in length), a small single-copy (SSC) region (16,726 bp in length), and two inverted repeat regions (IRs, 25,809 bp in length). The G + C content was 37.70% for the whole chloroplast and 42.88% for the IRs, which was higher than that of the LSC and SSC regions (35.61% and 31.90%, respectively). A total of 128 genes were found in the whole chloroplast genome, including 83 protein-coding genes, 37 tRNA genes, and eight rRNA genes.

**Figure 3.**
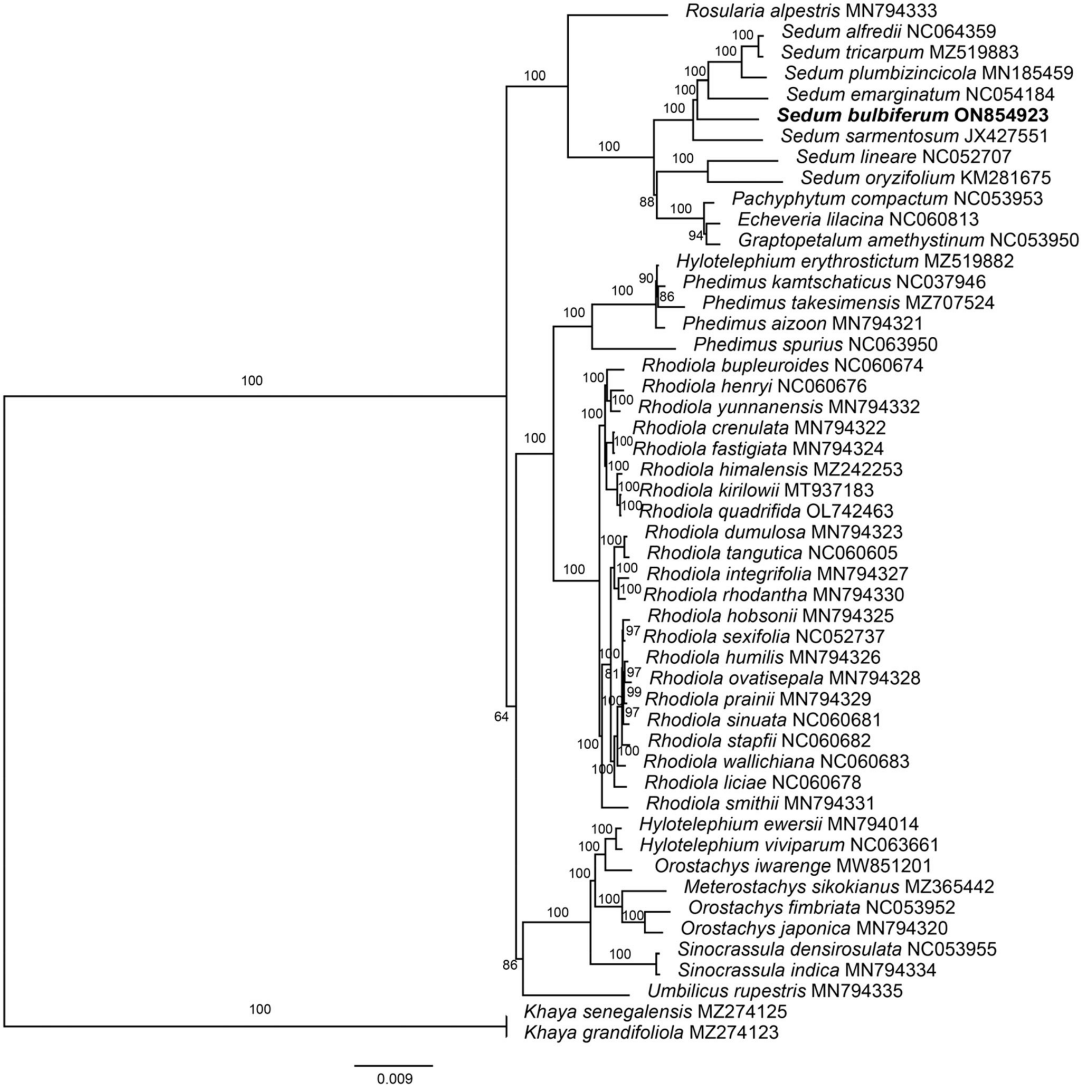
Maximum-likelihood (ML) tree of *S. bulbiferum* and 49 relative species was reconstructed using the IQ-Tree based on 55 protein-coding gene sequences shared by all genomes under the TVM + I + F model for 5000 ultrafast bootstraps, as well as the Shimodaira–Hasegawa–like approximate likelihood-ratio test. Bootstrap values are shown next to the nodes. The following sequences were used: *Rosularia alpestris* MN794333 (Zhao et al. 2020), *Sedum tricarpum* MZ519883 (Chen et al., 2022), *Sedum plumbizincicola* MN185459 (Ding et al. 2019), *Sedum lineare* NC052707 (Tang et al. 2021), *Sedum oryzifolium* KM281675 (Li and Chen 2020), *Echeveria lilacina* NC060813 (Nah et al. 2022), *Hylotelephium erythrostictum* MZ519882 (Chen et al., 2022), *Phedimus kamtschaticus* NC037946 (Seo and Kim 2018), *Phedimus takesimensis* MZ707524 (Seo et al. 2020), *Phedimus aizoon* MN794321 (Zhao et al. 2020), *Rhodiola bupleuroides* NC060674 (Zhao et al. 2021), *Rhodiola henryi* NC060676 (Zhao et al. 2021), *Rhodiola yunnanensis* MN794332 (Zhao et al. 2021), *Rhodiola crenulata* MN794322 (Zhao et al. 2020), *Rhodiola fastigiata* MN794324 (Zhao et al. 2020), *Rhodiola kirilowii* MT937183 (Zhang and Liu 2021), *Rhodiola quadrifida* OL742463 (Zhao et al. 2022), *Rhodiola dumulosa* MN794323 (Zhao et al. 2020), *Rhodiola tangutica* NC060605 (Lakshmanan et al. 2011), *Rhodiola integrifolia* MN794327 (Zhao et al. 2020), *Rhodiola rhodantha* MN794330 (Zhao et al. 2020), *Rhodiola hobsonii* MN794325 (Zhao et al. 2020), *Rhodiola humilis* MN794326 (Zhao et al. 2020), *Rhodiola ovatisepala* MN794328 (Zhao et al. 2020), *Rhodiola prainii* MN794329 (Zhao et al. 2020), *Rhodiola sinuata* NC060681 (Zhao et al. 2021), *Rhodiola stapfii* NC060682 (Zhao et al. 2021), *Rhodiola wallichiana* NC060683 (Zhao et al. 2021), *Rhodiola liciae* NC060678 (Zhao et al. 2021), *Rhodiola smithii* MN794331 (Zhao et al. 2020), *Hylotelephium ewersii* MN794014 (Zhao et al. 2020), *Meterostachys sikokianus* MZ365442 (Kang et al. 2022), *Orostachys japonica* MN794320 (Zhao et al. 2020), *Sinocrassula indica* MN794334 (Zhao et al. 2020), *Umbilicus rupestris* MN794335 (Zhao et al. 2020), *Khaya grandifoliola* MZ274123 (Mascarello et al. 2021), *Khaya grandifoliola* MZ274125 (Mascarello et al. 2021).

The phylogenetic analysis (Figure 3) showed that *S. emarginatum, S. alfredii, S. tricarpum, S. plumbizincicola,* and *S. sarmentosum* were closely related to *S. bulbiferum,* which is generally consistent with previous studies (Messerschmid et al. 2020).

## Discussion and conclusion

In this study, the whole chloroplast genome of *S. bulbiferum* was reported, and the phylogenic analysis results agreed with previous studies. However, due to the lack of the chloroplast genome of *Sedum* in public databases, the phylogeny of this genus requires further study. This study would provide a basis for the development of genetic resources and the evolutionary relationship of *Sedum*.

## Supplementary Material

Supplemental Material

## Data Availability

The complete chloroplast genome sequence of *Sedum bulbiferum* has been deposited in the GenBank database under the accession number ON854923 (https://www.ncbi.nlm.nih.gov/nuccore/ON854923). The associated BioProject, SRA, and Bio-Sample numbers are PRJNA867513, SRR20981864, and SAMN30203515, respectively.
