# Supplementary material for: The complete chloroplast genome sequence of Sedum bulbiferum (Crassulaceae)
Source: Mitochondrial DNA B Resour. 2023 May 24;8(5):598–602. doi: 10.1080/23802359.2022.2160220 (PMC10210845; doi:10.1080/23802359.2022.2160220)
Supplement: Supplemental Material [file TMDN_A_2160220_SM7220.docx]

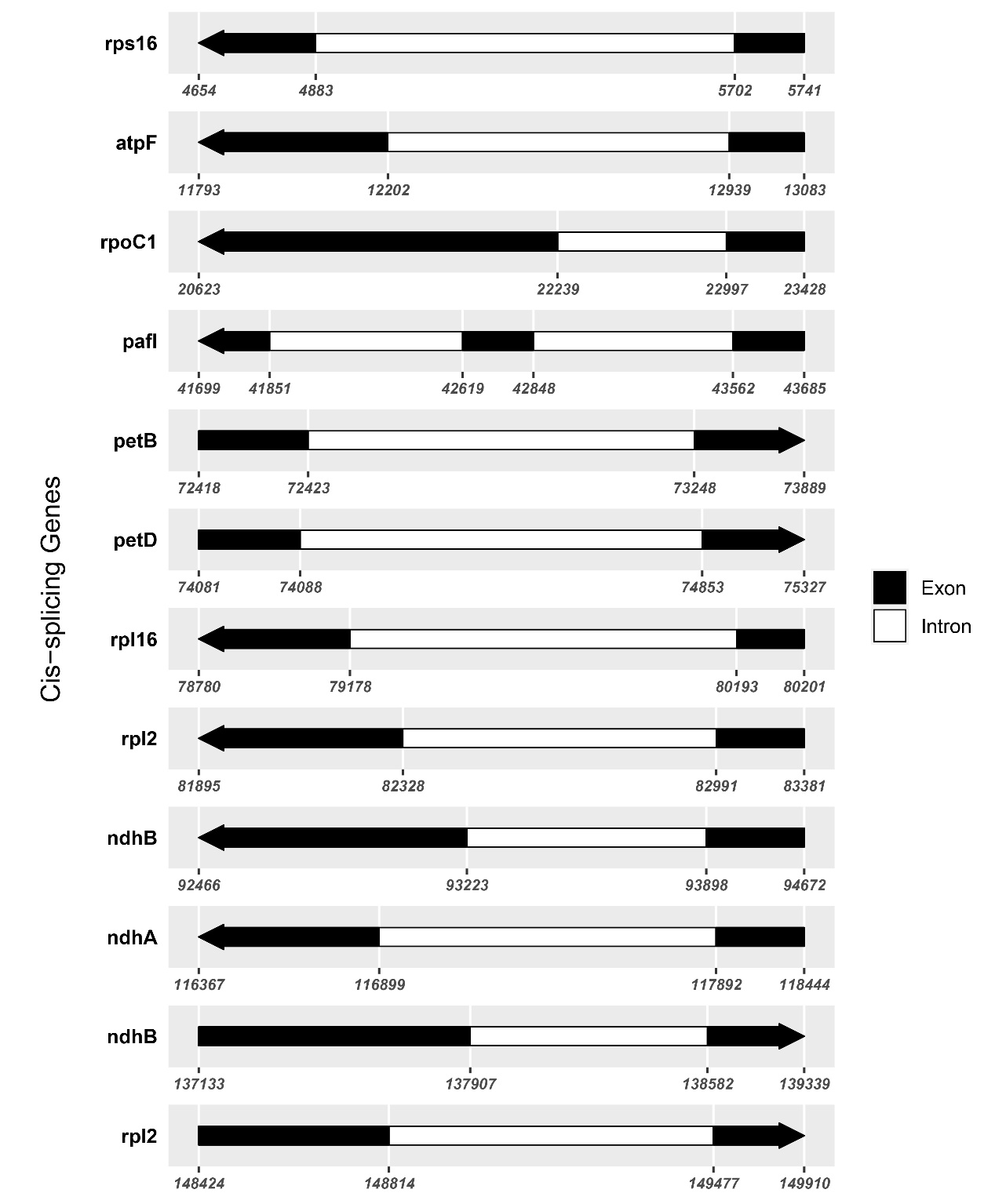


Supplemental figure 1. Schematic map of the cis-splicing genes in the chloroplast genome. The exons are shown in black; the introns are shown in white. The arrow indicates the sense direction of the gene. Please note that lengths of exons and introns are not drawn to scale.


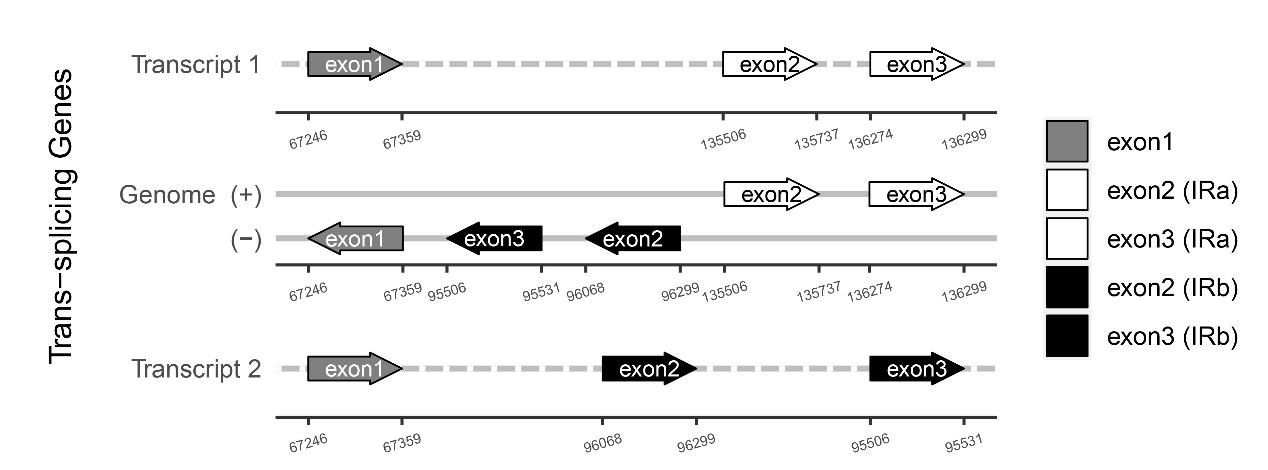


Supplemental figure 2. Schematic map of the trans-splicing gene rps12 in the chloroplast genome.
